# The evaluation of novel oral vaccines based on self-amplifying RNA lipid nanparticles (saRNA LNPs), saRNA transfected *Lactobacillus plantarum* LNPs, and saRNA transfected *Lactobacillus plantarum* to neutralize SARS-CoV-2 variants alpha and delta

**DOI:** 10.1038/s41598-021-00830-5

**Published:** 2021-10-29

**Authors:** Reza Keikha, Seyed Mohammad Hashemi-Shahri, Ali Jebali

**Affiliations:** 1 Infectious Diseases and Tropical Medicine Research Center, Resistant Tuberculosis Institute, Zahedan University of Medical Sciences, Zahedan, Iran; 2 Department of Pathology, Faculty of Medicine, Zahedan University of Medical Sciences, Zahedan, Iran; 3 Department of Medical Nanotechnology, Faculty of Advanced Sciences and Technology, Tehran Medical Science, Islamic Azad University, Tehran, Iran

**Keywords:** Immunology, Vaccines, RNA vaccines

## Abstract

The aim of this study was to present and evaluate novel oral vaccines, based on self-amplifying RNA lipid nanparticles (saRNA LNPs), saRNA transfected *Lactobacillus plantarum* LNPs, and saRNA transfected *Lactobacillus plantarum*, to neutralize severe acute respiratory syndrome coronavirus 2 (SARS-COV-2) variants alpha and delta. After invitro evaluation of the oral vaccines on HEK293T/17 cells, we found that saRNA LNPs, saRNA transfected *Lactobacillus plantarum* LNPs, and saRNA transfected *Lactobacillus plantarum* could express S-protein at both mRNA and protein levels. In the next step, BALB/c mice were orally vaccinated with saRNA LNPs, saRNA transfected *Lactobacillus plantarum* LNPs, and saRNA transfected *Lactobacillus plantarum* at weeks 1 and 3. Importantly, a high titer of IgG and IgA was observed by all of them, sharply in week 6 (P < 0.05). In all study groups, their ratio of IgG2a/IgG1 was upper 1, indicating Th1-biased responses. Wild-type viral neutralization assay showed that the secreted antibodies in vaccinated mice and recovered COVID-19 patients could neutralize SARS-COV-2 variants alpha and delta. After oral administration of oral vaccines, biodistribution assay was done. It was found that all of them had the same biodistribution pattern. The highest concentration of S-protein was seen in the small intestine, followed by the large intestine and liver.

## Introduction

Although different vaccines with different formulations have been developed against severe acute respiratory syndrome coronavirus 2 (SARS-COV-2), scientists are working on new vaccines with new formulations ^1^. The use of DNA/RNA is very promising technology because this is high-tech and can be well used in vaccine design ^2^. Although early studies focused on the use of DNA instead of RNA, the effectiveness of DNA-based vaccines was lower than RNA-based vaccines ^3^. Interestingly, RNA molecules are more unstable but they are more efficient invitro and invivo, especially when used together with nanoparticles ^4^. The reason of this phenomenon is that the functional site of RNA molecules is cytoplasm and they do not need to enter into the cell nucleus. The high efficacy of RNA vaccines has also been reported in cancer immunotherapy ^5,6^. There are two types of RNA vaccines, including messenger RNA (mRNA) and self-amplifying RNA (saRNA) ^7^. Although the design of RNA vaccines is a bit complicated, its scale up is not complicated ^8^. In addition to the antigen-encoding sequence, the sequence of RNA dependent RNA polymerase (RdRp) must also be inserted in the saRNA construct. The presence of RdRp leads to saRNA amplification in the cytoplasm ^9^. Due to unique property of saRNA, high immune responses can be achieved with low doses ^10^. Apart from the complex design of saRNA, its synthesis is no hard ^8^. After synthesizing of saRNA, the nanostructure carriers, such as cationic polymers ^11^, mannosylated lipid nanoparticles (MLNPs) ^12^, and eutral lipopolyplexes ^13^, are required to cross it into the cell membrane. Generally, saRNA vaccine are safe and there is no risk of DNA integration ^5,6,14^.

Although researchers are looking to design oral vaccines against SARS-COV-2, there is no approved oral vaccine yet ^15^. The design of oral vaccine is not simple and it has some challenges. For example, the enzymes of gastrointestinal tract (GIT) and the acidic environment of the stomach may break down the active components of vaccines ^16^. The next issue is that the viral genes may be not expressed in GIT cells. The antigenic proteins must be analyzed by the mucosal immune system. M cells deliver the antigenic proteins to the antigen-presenting cells (APCs) and then antigenic epitopes are expressed on the surface of APCs. Next, T cells and B cells will be activated by APCs. The activated immune cells will go to the mesenteric lymph nodes and they will produce secretory immunoglobulin A (S IgA) after transformation of B cells to plasma cells ^17^.

The aim of this study was to introduce and evaluate novel oral vaccines, based on saRNA LNPs, saRNA transfected *Lactobacillus plantarum* LNPs, and saRNA transfected *Lactobacillus plantarum* alone to neutralize SARS-CoV-2 variants alpha and delta.

## Results

### The characterization of saRNA construct and LNPs

**Figure 1:**
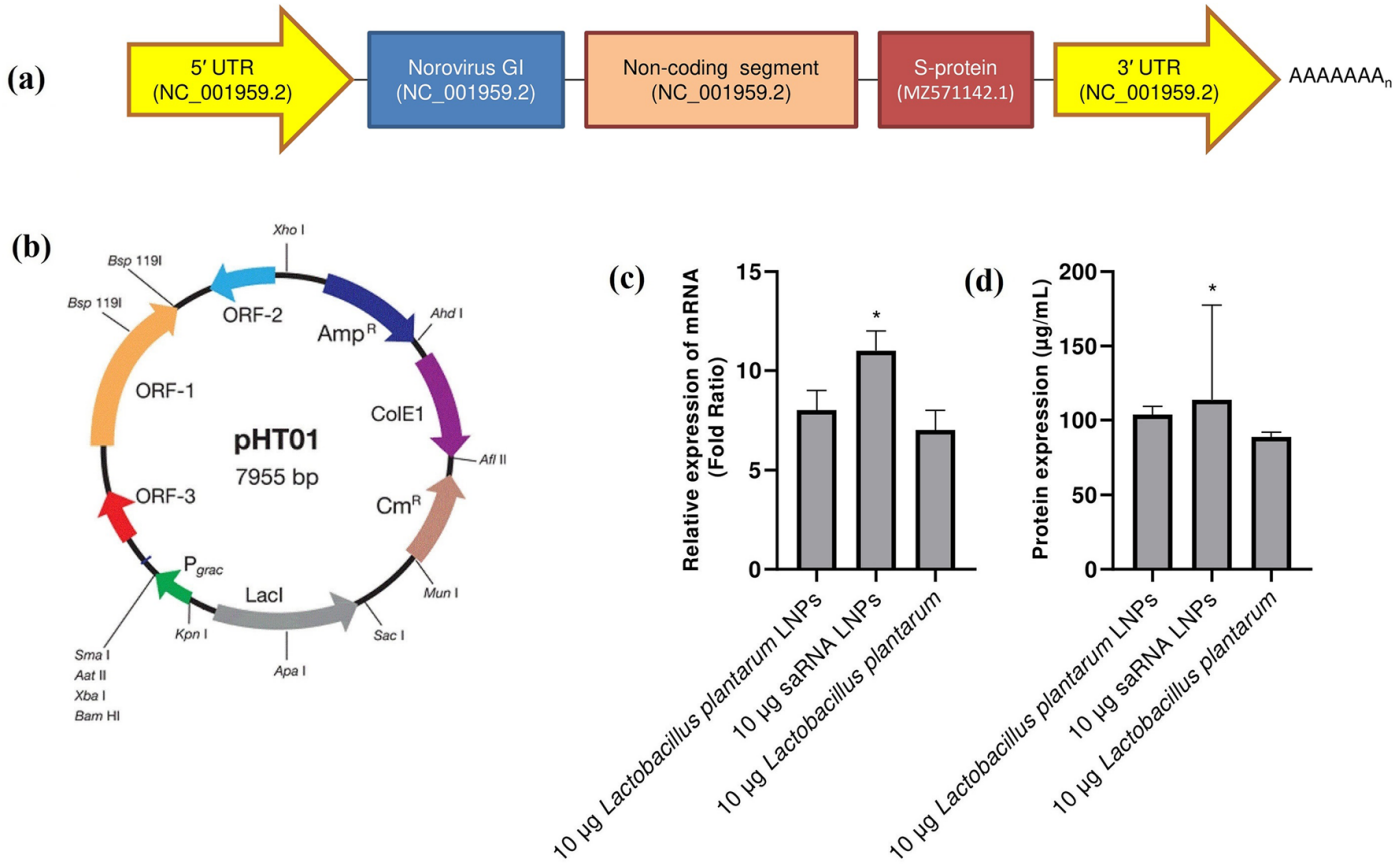
The schematic diagram of saRNA construct (**a**) which has different parts, including (1) 5′ UTR (GenBank accession number: NC_001959.2), (2) Norovirus GI (GenBank accession number: NC_001959.2), (3) noncoding segment (GenBank accession number: NC_001959.2), (4) S-protein (GenBank accession number: MZ571142.1), (5) 3′ UTR (GenBank accession number: NC_001959.2), and (6) polyA tail. The pHT01 plasmid map (https://www.addgene.org/vector-database/5885/) (**b**). HEK293T/17 cells were treated with saRNA LNPs, transfected *Lactobacillus plantarum* LNPs, and transfected *Lactobacillus plantarum* alone and the expression of S-protein was evaluated by real-time PCR (**c**) and ELISA (**d**). All of them could express S-protein at both mRNA and protein level. All data were shown as mean±SD, n = 3. * indicates significance difference with *P*<0.05 when compared with other formulations using one-way ANOVA. The limit of detection for ELISA was 0.1 µg/mL.

The saRNA construct (Fig. 1a) has different parts, including: (1) 5′ UTR (GenBank accession number: NC_001959.2), (2) Norovirus GI (GenBank accession number: NC_001959.2), (3) noncoding segment (GenBank accession number: NC_001959.2), (4) S-protein (GenBank accession number: MZ571142.1), (5) 3′ UTR (GenBank accession number: NC_001959.2), and (6) polyA tail. Figure 1b shows pHT01 plasmid map (https://www.addgene.org/vector-database/5885/). Both saRNA LNPs and negative control had approximately similar characteristics (Table 1). As seen, *Lactobacillus plantarum* LNPs had the bigger size distribution and less zeta potential.

**Table 1:** The characterization of LNPs used in this study.

	Particle size (nm)	Zeta potential (mV)	PDI
saRNA LNPs	500 ± 25	+ 17 ± 0.6	0.16 ± 0.03
*Lactobacillus plantarum* LNPs	1100 ± 6	+ 11 ± 0.6	0.18 ± 0.03
Negative control LNPs	500 ± 30	+ 16 ± 0.6	0.17 ± 0.03

### The invitro expression of S-protein

Before immunization of mice, HEK293T/17 cells were treated with saRNA LNPs, saRNA transfected *Lactobacillus plantarum* LNPs, and saRNA transfected *Lactobacillus plantarum* alone and the expression of S-protein was evaluated by real-time PCR and ELISA. We found that all treated cells could express S-protein at both mRNA (Fig. 1c) and protein (Fig. 1d) levels. Interestingly, the expression level of S-protein was significantly higher in HEK293T/17 cells treated with saRNA LNPs compared with transfected *Lactobacillus plantarum* LNPs or transfected *Lactobacillus plantarum* alone (P < 0.05).

### The antibody titer

**Figure 2:**
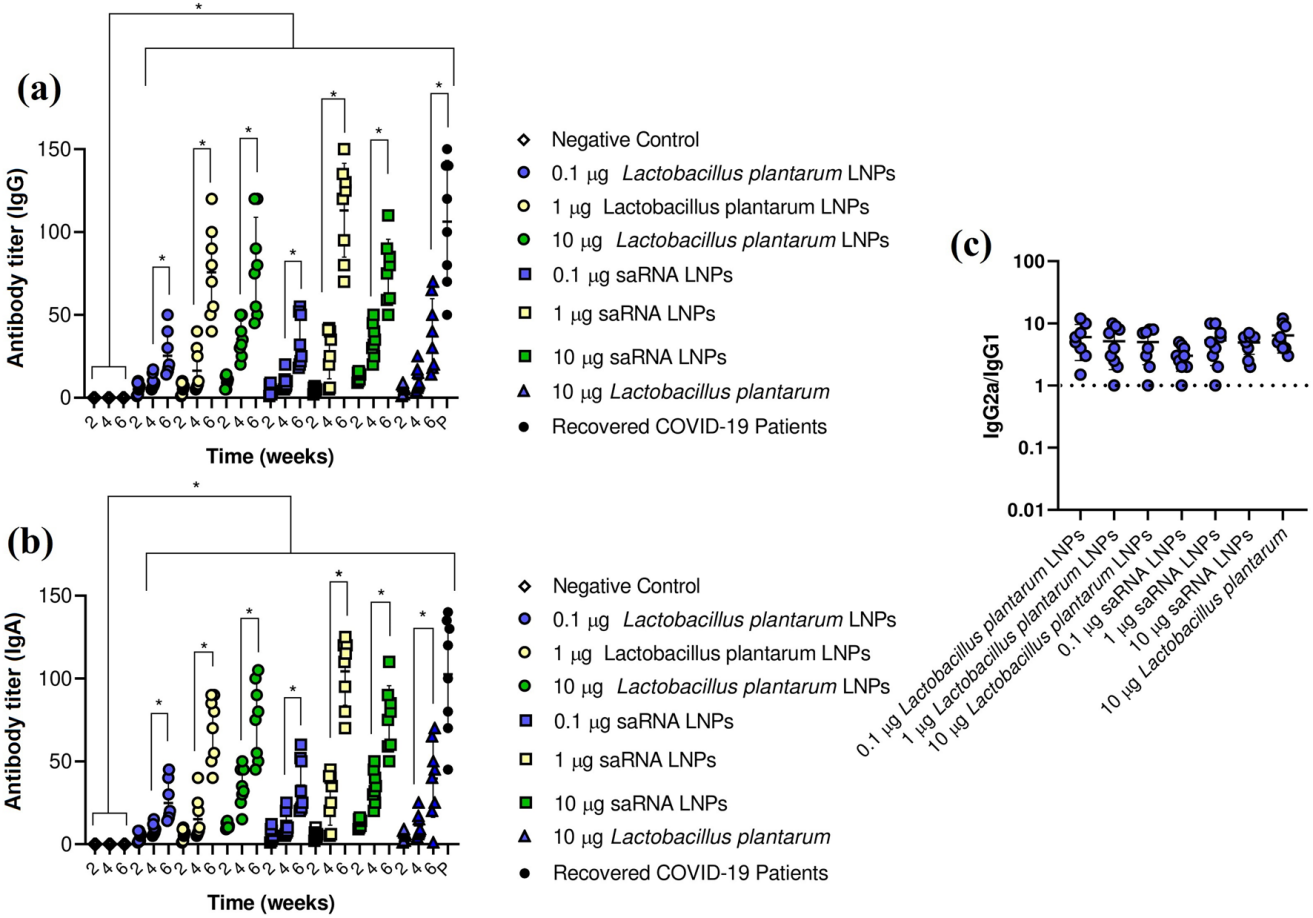
The IgG (**a**) and IgA (**b**) titer in mice vaccinated with saRNA LNPs, transfected *Lactobacillus plantarum* LNPs, and transfected *Lactobacillus plantarum* alone and in recovered COVID-19 patients against SARS-CoV-2. To find Th1-biased responses in vaccinated mice, the ratio of IgG2a/IgG1 was calculated (**c**). * indicates significance difference at *P* < 0.05 by one-way ANOVA with n = 10 biologically independent mice and n = 10 recovered COVID-19 patients. All data were shown as mean ± SD.

After immunization of BALB/c mice with saRNA LNPs, saRNA transfected *Lactobacillus plantarum* LNPs, and saRNA transfected *Lactobacillus plantarum* alone, a high titer of IgG (Fig. 2a) and IgA (Fig. 2b) was observed, sharply in week 6 (P < 0.05). Interestingly, the antibody titer of recovered COVID-19 patients was comparable with antibody titer of vaccinated mice at week 6 (P > 0.05). Another finding was that the production of antibody in vaccinated mice was dose and time dependent. To find Th1-biased responses in vaccinated mice, the ratio of IgG2a/IgG1 and was measured. In all study groups, their ratio of IgG2a/IgG1 was upper 1, indicating a Th1-biased response (Fig. 2c).

### Viral neutralization assay

**Figure 3:**
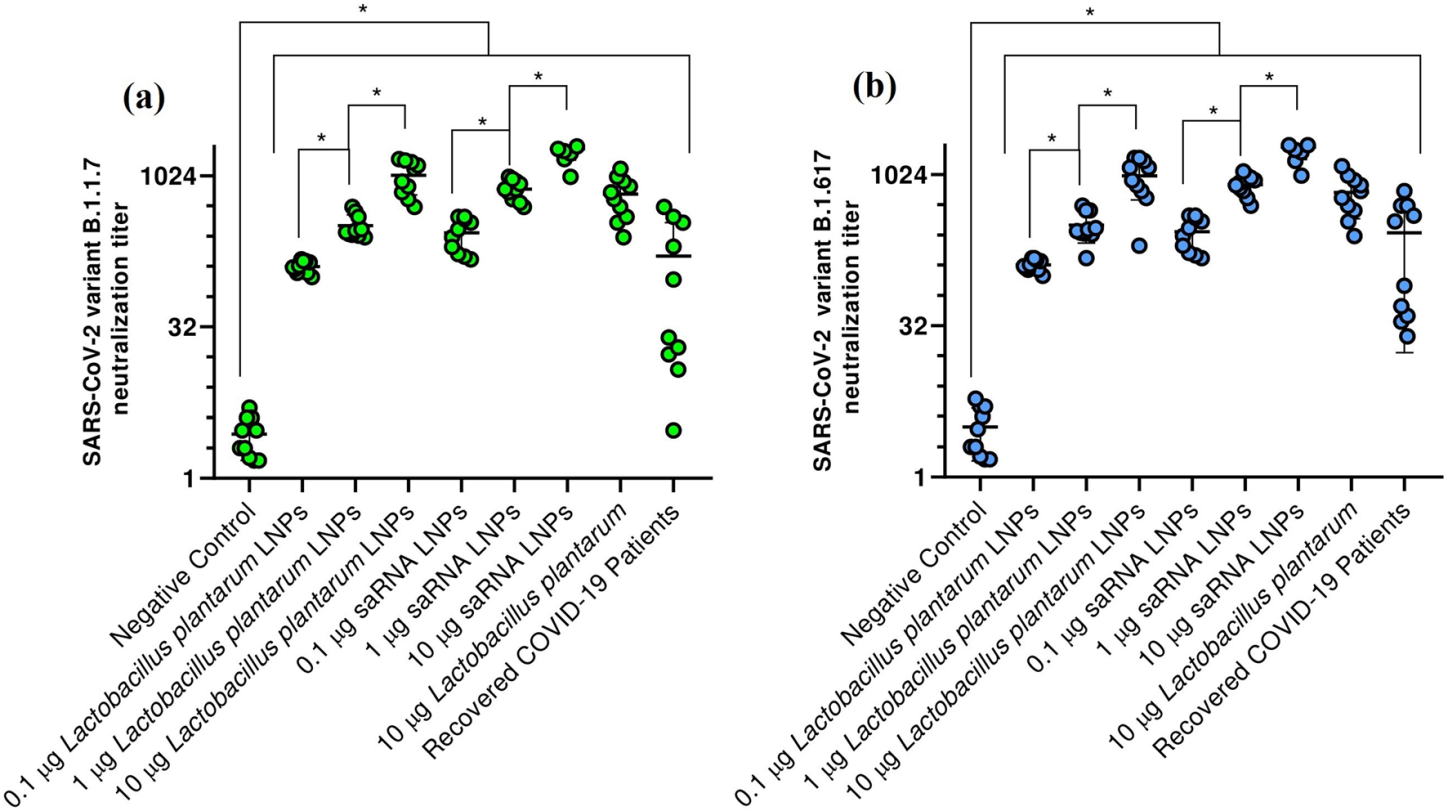
The viral neutralization titer against SARS-CoV-2 variants B.1.1.7 (**a**) and B.1.617 (**b**) in mice vaccinated with saRNA LNPs, transfected *Lactobacillus plantarum* LNPs, and transfected *Lactobacillus plantarum* alone and in recovered COVID-19 patients. * indicates significance difference with *P* < 0.05 using one-way ANOVA with n = 10 biologically independent mice and n = 10 recovered COVID-19 patients. All data were shown as mean ± SD.

The secreted antibodies in vaccinated mice and recovered COVID-19 patients could neutralize SARS-COV-2 variants alpha (B.1.1.7) (Fig. 3a) and delta (B.1.617) (Fig. 3b). There were significant differences between the neutralization titer of all study groups and negative control (P < 0.05). Also, there were significant differences between neutralization titer in mice vaccinated with different dose of saRNA LNPs and transfected *Lactobacillus plantarum* LNPs (P < 0.05). Importantly, there was no significant difference between different formulation at the same dose (P > 0.05). The correlation analysis between SARS-CoV-2 specific IgG or IgA titer and SARS-CoV-2 neutralization titer can be seen in Supplementary 1.

### IFN-γ ELISpots and the secretion of IL-6

**Figure 4:**
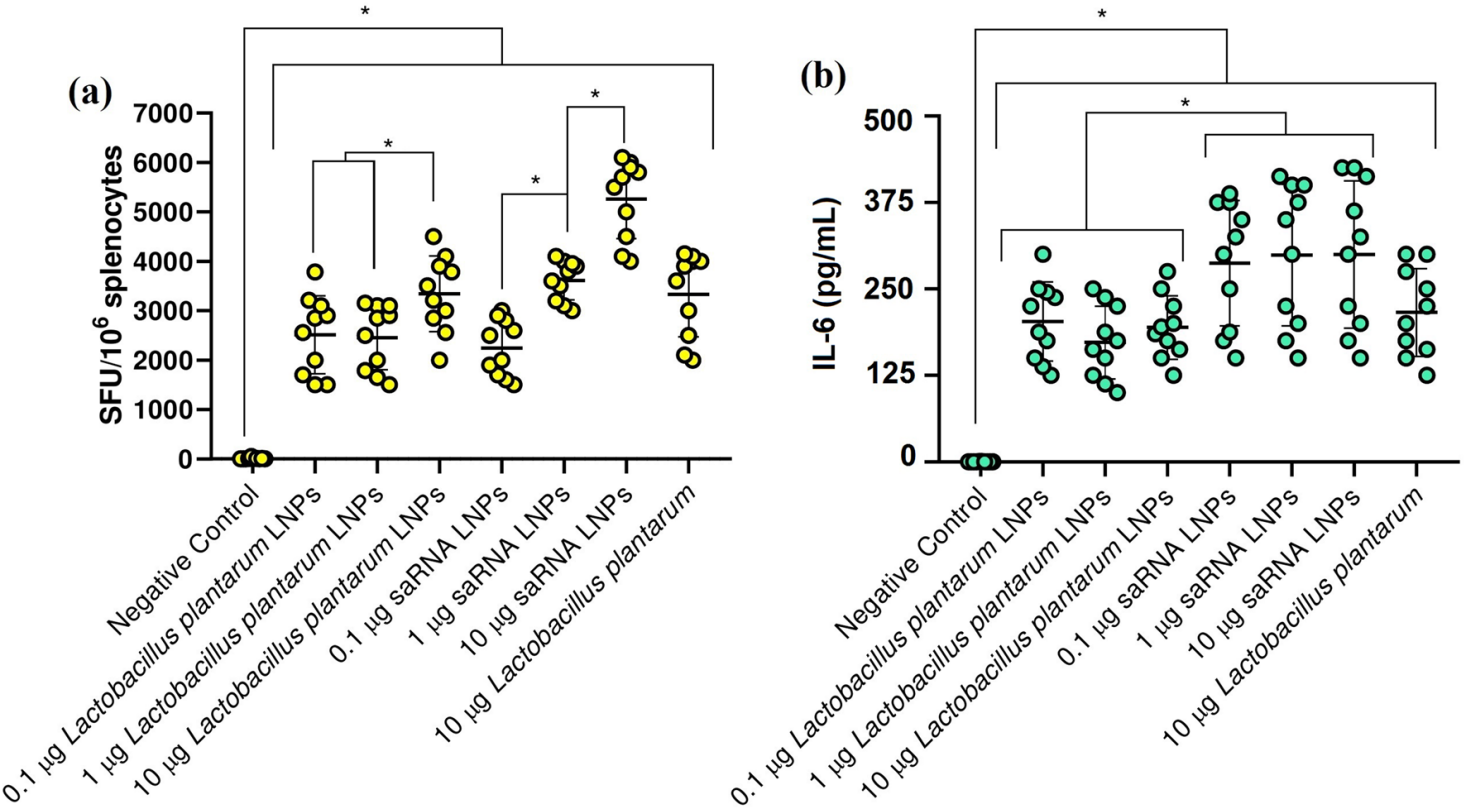
The count of splenocytes from mice vaccinated with saRNA LNPs, transfected *Lactobacillus plantarum* LNPs, and transfected *Lactobacillus plantarum* alone when re-stimulated with SARS-CoV-2 peptides by IFN-γ ELISpots (**a**). The secretion of IL-6 (**b**) by re-stimulated splenocytes. * indicates significance difference with *P* < 0.05 using one-way ANOVA with n = 10 biologically independent mice. All data were shown as mean ± SD.

Splenocytes from mice immunized with saRNA LNPs, saRNA transfected *Lactobacillus plantarum* LNPs, and saRNA transfected *Lactobacillus plantarum* alone had a high IFN-γ secretion with dose-dependent manner (Fig. 4a). Significant differences were seen between splenocytes count in all immunized mice and negative control (P < 0.05). The secretion of IL-6 (Fig. 4b) by re-stimulated splenocytes confirmed this data. Significant differences were also seen between the concentration of IL-6 produced by activated splenocytes in all immunized mice and negative control (P < 0.05). We also analyzed the serum level of IFN-γ, TNF-α, IL-4, and IL-10 in all vaccinated mice (supplementary 2).

### Biodistribution assay

**Figure 5:**
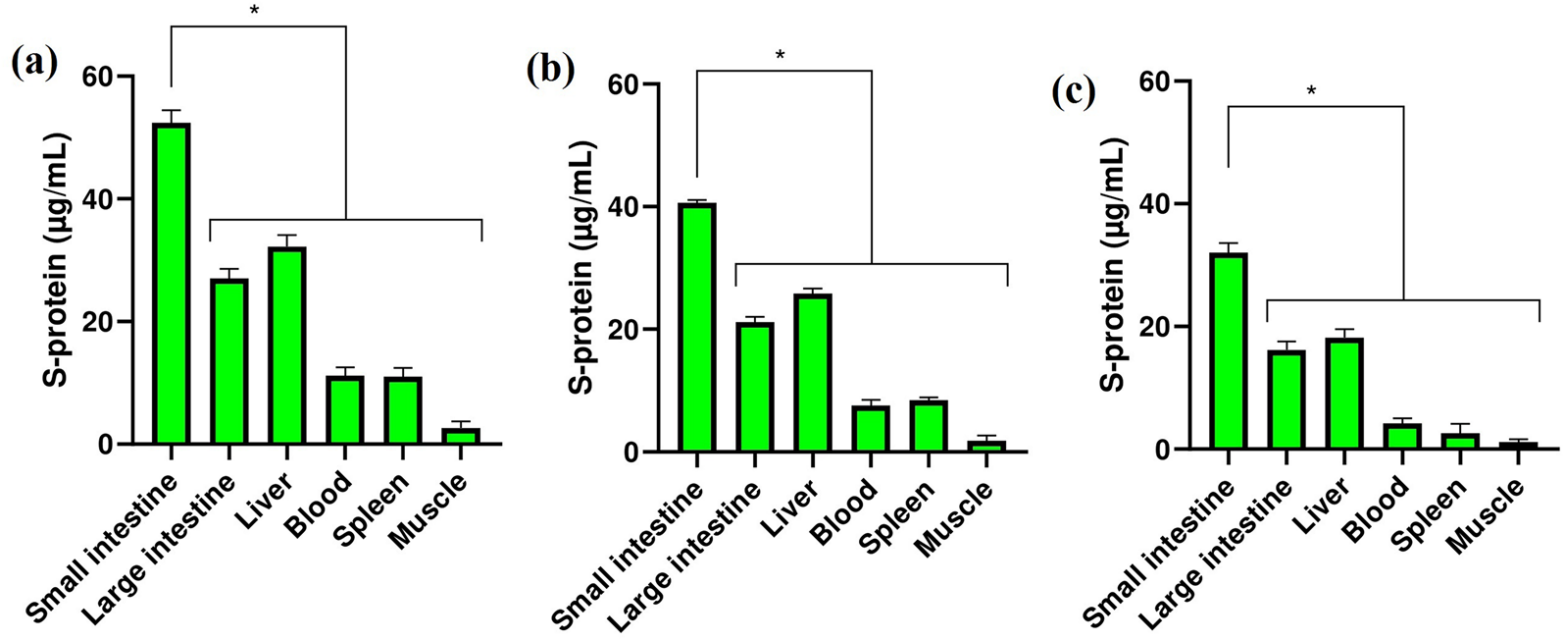
The level of S-protein in major organ or tissues of mice vaccinated with saRNA LNPs (**a**), transfected *Lactobacillus plantarum* LNPs (**b**), and transfected *Lactobacillus plantarum* alone (**c**). * indicates significance difference with *P* < 0.05 using one-way ANOVA with n = 10 biologically independent mice. All data were shown as mean ± SD.

Biodistribution assay showed that saRNA LNPs (Fig. 5a), saRNA transfected *Lactobacillus plantarum* LNPs (Fig. 5b), and saRNA transfected *Lactobacillus plantarum* alone (Fig. 5c) had the same biodistribution pattern. The highest concentration of S-protein was seen in the small intestine, followed by the large intestine and liver. Significant differences was seen between the concentration of S-proein in small intestine and other organs (P < 0.05).

## Discussion

In this project, a saRNA construct with the sequence of Norovirus GI (GenBank accession number: NC_001959.2) and the sequence of Spike protein of SARS-CoV-2/human/MAR/CNRST-IND2-2021/2021 was sub-cloned in the pHT01 plasmid. In the next step, linear saRNA was synthesized and BALB/c mice were orally vaccinated by different dose of saRNA LNPs. We found that saRNA LNPs had high efficacy to produce IgG and IgA to neutralize SARS-COV-2 variants B.1.1.7 and B.1.617. McKay et al. had been previously shown that saRNA SARS-CoV-2 LNP vaccine induces remarkably high and dose-dependent SARS-CoV-2 specific antibody titers in mouse ^18^. Also, Spencer et al. showed the vaccination with saRNA and adenoviral vaccines induced robust immune responses in mice ^19^. In our study, BALB/c mice were also orally vaccinated by saRNA transfected *Lactobacillus plantarum* LNPs and saRNA transfected *Lactobacillus plantarum* alone. Miraculously, we saw that both of them could also stimulate the immune system of mice, produce IgG and IgA, and neutralize SARS-COV-2. In this study, we encapsulated saRNA molecules and transfected *Lactobacillus plantarum* by LNPs to escape the turbulent GIT environment. If we wanted to make a comparison and say which formulation had higher efficacy, we can say that saRNA LNPs was better than other formulation at the same dose. The saRNA transfected *Lactobacillus plantarum* LNPs and saRNA transfected *Lactobacillus plantarum* alone were in the next level. Based on biodistribution assay, saRNA LNPs, saRNA transfected *Lactobacillus plantarum* LNPs, and saRNA transfected *Lactobacillus plantarum* alone had the same biodistribution pattern and the highest concentration of S-protein was seen in mice vaccinated with saRNA LNPs. It seems that all the formulations used in this study were able to pass through the stomach and reach to small and large intestines. It is a very valuable finding because these formulations may be used as an edible vaccine. The term of edible vaccines was first used by Charles Arendzen in 1990 to refer to any type of food that can stimulate the immune system and act as a vaccine against a specific disease ^20^. In general, when a food product is ingested by GIT, both mucosal and humoral immune systems are stimulated ^21^. Edible vaccines are genetically modified products that have an immunogenic component to produce antibody ^22^. They have many advantages compared with traditional vaccines, such as lower manufacturing cost and side effects ^23^. The production of traditional vaccines is very expensive and limited in most countries, but in contrast, the production, purification, sterilization, and distribution of edible vaccines are easier ^24^. However, they have also some limitations because edible vaccines are still new and in development and more researches must be done before widespread human usage ^21^. In 2006, Lee et al. immunized mice by *Lactobacillus casei* expressed S-protein of SARS. The immunization led to the high production of IgG and IgA against SARS ^25^. In 2020, Wang et al. engineered a transgenic strain of *Lactobacillus plantarum* that expressed S-protein of SARS-COV-2. They showed that S-protein was effectively expressed by transfected *Lactobacillus plantarum*. Importantly, the S-protein expressed in *Lactobacillus plantarum* was stable at 50 °C, pH = 1.5 ^26^.

Like all experimental studies, this study had some limitations. Here, there was no estimation on the durability of responses for antibodies and T-cell response. Also, there is no head to head comparison with "traditional" mRNA vaccine. These limitations must be considered in future studies. On the other hands, this study had significant scientific novelties, including: (1) the use of Norovirus sequence in the saRNA construct. Norovirus has two main structural proteins which can bind to different cells of gut ^27^. Also, human norovirus also targets enteroendocrine epithelial cells in the small intestine ^28^. It is the first report for COVID-19 vaccine and this formulation can be used for both oral and edible vaccine. (2) The use of saRNA transfected *Lactobacillus plantarum* LNPs and saRNA transfected *Lactobacillus plantarum* alone as novel oral vaccines against SARS-COV-2. It is the first report and this finding can help us to design edible vaccines against SARS-COV-2. In conclusion, the oral vaccines, based on saRNA LNPs, saRNA transfected *Lactobacillus plantarum* LNPs, and saRNA transfected *Lactobacillus plantarum* alone, could induce a Th1-biased response to produce a high titer of IgG and IgA against SARS-CoV-2. The produced antibodies could neutralize SARS-CoV-2 variants alpha and delta. An important finding was that all formulations had the same biodistribution pattern and the highest concentration of S-protein was seen in small intestine of mice vaccinated with saRNA LNPs.

## Materials and methods

### Plasmid construct

To design a plasmid construct for saRNA, pHT01 as a common bacterial plasmid with a T7 promoter, was used. The full data of plasmid can be seen at https://www.addgene.org/vector-database/5885/. The sequence of Norovirus GI (GenBank accession number: NC_001959.2) and the sequence of Spike protein of SARS-CoV-2/human/IND/AS-RMRC-233571/2021 (GenBank accession number: MZ149976) were the basic parts of saRNA construct. The sequence was synthesized, sub-cloned, and confirmed by Biomatik, Canada. The full length of saRNA construct can be seen in supplementary 3. In the negative control plasmid, there was no saRNA sequence.

### Bacterial transfection

According to the article of Wang et al. ^26^, 2 × 10^6^ CFU/mL *Lactobacillus plantarum* (ATCC: 8014) was transfected with saRNA plasmid by electroporation. The bacteria were then grown on kanamycin-containing medium and the transfected *Lactobacillus plantarum* were selected. After bacterial selection, they were cultured and their plasmids were purified by Plasmid Plus MaxiPrep kit (QIAGEN, UK). Then, 1 ng of purified plasmid, 0.5 μM primers (forward: 5′-CTATCAGGCCGGTAGCACAC-3′ and reverse: 5′-ACACCTGTGCCTGTTAAACCA-3′) and 2 Units of Mastermix DNA Polymerase (Thermo Fisher Scientific) were added and amplified under the following conditions: 35 cycles of 95 °C for 10 s, 55 °C for 30 s, and 72 °C for 10 s. Finally, the presence of saRNA plasmid was confirmed by agarose gel electrophoresis (supplementary 4).

### Synthesis of linear saRNA

The sequence required for linear saRNA was first cloned by PCR. For this purpose, 1 ng of purified plasmid, 0.5 μM primers (forward: 5′- GTGAATGATGATGGCGTC -3′ and reverse: 5′- TTTTTAACATCAAATTAA-3′) and 2 U of Mastermix PFU DNA Polymerase (Thermo Fisher Scientific) were added and amplified under the following conditions: 35 cycles of 95 °C for 10 s, 55 °C for 30 s, and 72 °C for 10 s. The cloned linear DNA was purified by a Plasmid Plus MaxiPrep kit (QIAGEN, UK) and its purity was determined by a NanoDrop (ThermoFisher, UK). Next, 10 ng purified DNA was first reacted with MEGAScript™ (Ambion, UK) for 1 h at 37 °C and then it was reacted with ScriptCap™ (CellScript, WI, USA) for 1 h at 37 °C. Then, synthesized linear saRNA was purified by LiCl precipitation, re-suspended in RNA storage buffer, and stored at − 80 °C. The purity of synthesized saRNA was finally checked by NanoDrop (ThermoFisher, UK) ^18^.

### Encapsulation of linear saRNA and transfected *Lactobacillus plantarum*

To encapsulate saRNA and transfected *Lactobacillus plantarum*, we used a simple chemical process ^18^ in which 100 1 µg purified saRNA and 10^6^ CFU/mL of transfected *Lactobacillus plantarum* were separately mixed with an ethanolic lipid mixture of 1,2-dilinoleyloxy-3-dimethylaminopropane, 1,2-diastearoylsn-glycero-3-phosphocholine, cholesterol, and PEG-DMG 2000 at a ratio of 10:48:2:40. The mixture was stirred vigorously by a T-mixer and then placed in a dialysis bag to purify overnight. The synthesized LNPs were stored at 4 °C. The particle size distribution, zeta potential, polydispersity index (PDI) of LNPs were determined by a dynamic light scattering (DLS) (Malvern Instruments Ltd, Malvern, UK). For negative control plasmid, the same encapsulation process was also carried out.

### The invitro expression of S-protein

HEK293T/17 cells were first obtained from institute Pasteur of Iran. Then, they were cultured in complete Dulbecco’s Modified Eagle’s Medium (DMEM) (Gibco) containing 10% fetal bovine serum (FBS) (Gibco), 1% L-glutamine (Thermo Fisher Scientific), and 1% penicillin–streptomycin (Thermo Fisher Scientific) at 37 °C and 5% CO^2^ ^18^. Then, 2 × 10^6^ cells/mL HEK293T/17 cells were separately treated with (1) 10 µg saRNA LNPs, (2) 10 µg transfected *Lactobacillus plantarum* LNPs, and (3) 10 µg transfected *Lactobacillus plantarum* for 5 days at 37 °C and 5% CO_2_. After incubation, the expression of S-protein was confirmed by q-PCR and ELISA (supplementary 5).

### Immunization and collecting serum samples

BALB/c mice aged 6–8 weeks were orally immunized with:

1. 0.1 µg saRNA LNPs at weeks 1 and 3.
2. 1 µg saRNA LNPs at weeks 1 and 3.
3. 10 µg saRNA LNPs at weeks 1 and 3.
4. 0.1 µg *Lactobacillus plantarum* LNPs at weeks 1 and 3.
5. 1 µg *Lactobacillus plantarum* LNPs at weeks 1 and 3.
6. 10 µg *Lactobacillus plantarum* LNPs at weeks 1 and 3.
7. 10 µg *Lactobacillus plantarum* at weeks 1 and 3.

In this study, vaccine administration was done by a needleless insulin syringe and the vaccine volume was 100 µL for each dose. Serum samples were collected at weeks 2, 4, and 6 and the spleens were removed at week 6. Here, the serum samples of recovered COVID-19 patients (n = 10, age = 40 ± 5, female/male = 60/40), suffered from SARS-COV-2 variant B.1.617 were also collected from Zahedan University of Medical Sciences, Zahedan, Iran following written informed consent (IR.ZAUMS.REC.1399.317 and IR.ZAUMS.REC.1399.316). All of them had grade II COVID-19 and they had been hospitalized for 2 weeks. One week after discharge from the hospital, Real-time PCR was done and their serum samples were collected. All recovered COVID-19 patients had negative PCR result at the time of sampling. Here, all experiments were under an approval of ethical committee of Zahedan University of Medical Sciences, Zahedan, Iran (IR.ZAUMS.REC.1400.071).

### The evaluation of antibody titer

To evaluate antibody titer, an ELISA assay was used, according to Tian et al. ^29^. In the first step, a high binding ELISA plates (Biomat, Italy) were coated with SARS-CoV-2 Spike Protein Recombinant Antigen (Sigma-Aldrich) at 1 mg/mL in PBS overnight at 4 °C. The plates were washed 3 times with PBS and blocked with 10% BSA (Sigma-Aldrich) and 3% sucrose (Sigma-Aldrich) at 4 °C overnight. Then, to inactivate blood complements, all serum samples were incubated at 56 °C for 30 min. The serum samples were separately diluted and 100 µL of each serum samples was added to the ELISA plate. The plates were incubated at 37 °C for 2 h and then were washed 3 times with PBS. After incubation and washing, they were separately incubated with secondary antibodies, including (1) anti-mouse and anti-human IgG-HRP (Southern Biotech), (2) anti-mouse IgG1-HRP (Southern Biotech), (3) anti-mouse IgG2a-HRP (Southern Biotech), (4) anti-mouse and anti-human IgA-HRP (Southern Biotech), with 1:5000 at 37 °C for one hour. After washing with PBS, 100 µL 3,3′, 5,5′-tetramethylbenzidine (TMB) substrate (Sigma-Aldrich) was added and incubated at 37 °C for 15 min. Then, 100 µL of sulfuric acid 1% (Sigma) was added and the optical density (OD) of each well was measured at 450 nm by a spectrophotometer (BioTek Industries). To set up ELISA cut-off values, 5 healthy mice without any vaccination and 5 healthy human which did not receive COVID-19 vaccine or suffered from COVID-19 were considered as negative control. The serum sample was considered positive when the OD was above the cut-off value. The cut-off value for mouse and human IgG was 0.140, for mouse and human IgA was 0.150, for mouse IgG2a and IgG1 was 0.155. Finally, the highest dilution titer which was above cut-off value was recorded.

### Wild-type viral neutralization assay

To evaluate the ability of sera to neutralize SARS-CoV-2 virus, wild-type viral neutralization assay was applied. Based on the article of McKey et al. ^18^, SARS-CoV-2 variant B.1.1.7 and variant B.1.617 were isolated and cultured on Caco2 cells in DMEM (Gibco) containing 10% FBS (Gibco), 1% L-glutamine (Thermo Fisher Scientific), and 1% penicillin–streptomycin (Thermo Fisher Scientific) for 5 days at 37 °C. These variants were from clinical samples from reference laboratory of Zahedan University of Medical Sciences, Zahedan, Iran. Finally, they were purified by Caesium chloride. The serum samples were taken from vaccinated mice at week 6 and all of them were first incubated at 56 °C for 30 min and were serially diluted in DMEM (Gibco, Thermo Fisher Scientific) with 1% penicillin–streptomycin (Thermo Fisher Scientific), 0.3% BSA fraction V (Thermo Fisher Scientific) and 0.25 µg/mL trypsin (Worthington). Serum dilutions were separately incubated with 100 TCID_50_ per well of SARS-CoV-2 variant B.1.1.7 and variant B.1.617 for 1 h at room temperature. Then, they were transferred to 96-well plates pre-seeded with HEK293T/17 cells and incubated at 37 °C for 5 days. After incubation, 100 µL of crystal violet (Sigma-Aldrich) was added to each well and scored the cytopathic effect. The neutralization titer was calculated as the reciprocal of the highest serum dilution at which full virus neutralization occurred.

### IFN-γ ELISpots and the secretion of IL-6

Anti-IFN-γ pre-coated plates (Mabtech) were first blocked with 10% FBS. Then, 2.5 × 10^5^ mouse splenocytes and 1 µg mL^−1^ SARS-CoV-2 peptide pools (15-mers overlapping by 11; JPT Peptides) were added and incubated overnight for 24 h at 37 °C and 5% CO_2_. After incubation, biotinylated cytokine-specific detection antibodies (Mabtech), streptavidin-enzyme conjugate, and substrate (Mabtech) were added. Finally, each well was observed under an optical microscope (Zeiss, Germany) and the number of secreting cells was calculated ^18,30^.

To detect the level of IL-6, a high binding ELISA plate (Biomat, Italy) was separately coated with anti-mouse IL-6 (Southern Biotech) and then diluted samples from supernatant of activated mouse splenocytes were separately added. After 1 h incubation at 37 °C, plates were washed with PBS and then 100 μL of secondary antibody, including anti-mouse IL-6-HRP (Southern Biotech), was added. Then, 50 μL of TMB (Sigma-Aldrich) was added. After 15 min, 100 μL sulfuric acid (Sigma) was added and OD of each well was read by a Spectrophotometer at 450 nm (BioTek Industries) and then the serum level of IL-6 was quantified by standard curve ^18^.

### Biodistribution assay

Biodistribution assay ^31^ was used to find out how saRNA LNPs, transfected *Lactobacillus plantarum* LNPs, and transfected *Lactobacillus plantarum* alone are distributed and expressed in different organs and tissue of mice. For this purpose, BALB/c mice aged 6–8 weeks were orally administered with 10 µg saRNA LNPs, 10 µg transfected *Lactobacillus plantarum* LNPs, and 10 µg transfected *Lactobacillus plantarum*. After 24 h, mice were sacrificed and sampled from their major organs and tissues, such as small intestine, large intestine, liver, blood, spleen, and muscle. Then, the concentration of S-protein was quantified by ELISA (supplementary 6).

### Statistical analysis

All graphs and data are shown as Mean ± SD. GraphPad Prism (version 8.4) was used to prepare graphs and statistics. Here, one-way ANOVA was used to indicate significance at *P* < 0.05 after post-hoc analyses, such as Tukey and Dunnett. In this study, 10 biological independent mice and 10 recovered COVID-19 patients were used for related experiments.

### Ethics approval for animal experiments

All experiments were under the guidelines of the National Institute of Health, the provisions of the Declaration of Helsinki, and the ethics committee of Zahedan University of Medical Sciences, Zahedan, Iran (Ethical code: IR.ZAUMS.REC.1400.071).

### Ethics approval for human experiments

The serum samples of recovered COVID-19 patients were collected from Zahedan University of Medical Sciences, Zahedan, Iran following written informed consent. Here, all experiments were under an approval of ethical committee of Zahedan University of Medical Sciences, Zahedan, Iran (IR.ZAUMS.REC.1399.317 and IR.ZAUMS.REC.1399.316).

### Author declarations

The authors confirm that all experiments were performed in accordance with ARRIVE guidelines and regulations of Zahedan University of Medical Sciences, Zahedan, Iran. We confirm that all experimental protocols were approved by Zahedan University of Medical Sciences, Zahedan, Iran. We confirm that all methods were carried out in accordance with relevant guidelines and regulations of Zahedan University of Medical Sciences, Zahedan, Iran. We confirm that all methods were reported in accordance with ARRIVE guidelines (https://arriveguidelines.org) for the reporting of animal experiments.

## Supplementary Information

Supplementary Information 1.

Supplementary Information 2.

Supplementary Information 3.

Supplementary Information 4.

Supplementary Information 5.

Supplementary Information 6.

## Data Availability

All data of this article is available based on the official request of researchers.
