# Supplementary material for: The evaluation of novel oral vaccines based on self-amplifying RNA lipid nanparticles (saRNA LNPs), saRNA transfected Lactobacillus plantarum LNPs, and saRNA transfected Lactobacillus plantarum to neutralize SARS-CoV-2 variants alpha and delta
Source: Sci Rep. 2021 Oct 29;11:21308. doi: 10.1038/s41598-021-00830-5 (PMC8556360; doi:10.1038/s41598-021-00830-5)
Supplement: Supplementary file 1 — Supplementary Information 1. [file 41598_2021_830_MOESM1_ESM.docx]

**Supplementary 1.** The correlation between SARS-CoV-2 specific IgG or IgA titer and SARS-CoV-2 neutralization titer

| **SARS-CoV-2 specific IgA titer** | **SARS-CoV-2 specific IgG titer** |  | |
| --- | --- | --- | --- |
| R=0.87, *P=*0.02 | R=0.88, *P=*0.01a | 0.1 μg *Lactobacillus plantarum* LNPs | **SARS-CoV-2 variant B.1.1.7 neutralization titer** |
| R=0.89, *P=*0.001 | R=0.90, *P=*0.001 | 1 μg *Lactobacillus plantarum* LNPs |  |
| R=0.93, *P=*0.0001 | R=0.95, *P=*0.0001 | 10 μg *Lactobacillus plantarum* LNPs |  |
| R=0.86, *P=*0.03 | R=0.89, *P=*0.01 | 0.1 μg saRNA LNPs |  |
| R=0.90, *P=*0.001 | R=0.92, *P=*0.001 | 1 μg saRNA LNPs |  |
| R=0.94, *P=*0.0001 | R=0.96, *P=*0.0001 | 10 μg saRNA LNPs |  |
| R=0.85, *P=*0.03 | R=0.87, *P=*0.02 | 10^6^ CFU/mL of *Lactobacillus plantarum* |  |
| R=0.79, *P=*0.02 | R=0.80, *P=*0.01 | Recovered COVID-19 Patients |  |
| R=0.81, *P=*0.01 | R=0.82, *P=*0.03 | 0.1 μg *Lactobacillus plantarum* LNPs | **SARS-CoV-2 variant B.1.617 neutralization titer** |
| R=0.88, *P=*0.003 | R=0.91, *P=*0.001 | 1 μg *Lactobacillus plantarum* LNPs |  |
| R=0.90, *P=*0.0001 | R=0.93, *P=*0.0001 | 10 μg *Lactobacillus plantarum* LNPs |  |
| R=0.86, *P=*0.02 | R=0.85, *P=*0.02 | 0.1 μg saRNA LNPs |  |
| R=0.89, *P=*0.002 | R=0.90, *P=*0.002 | 1 μg saRNA LNPs |  |
| R=0.92, *P=*0.0001 | R=0.94, *P=*0.0001 | 10 μg saRNA LNPs |  |
| R=0.82, *P=*0.01 | R=0.84, *P=*0.03 | 10^6^ CFU/mL of *Lactobacillus plantarum* |  |
| R=0.75, *P=*0.03 | R=0.79, *P=*0.02 | Recovered COVID-19 Patients |  |

^a^ The significant direct correlations were observed between SARS-CoV-2 specific IgG or IgA titer and SARS-CoV-2 neutralization titer
