# Supplementary material for: The evaluation of novel oral vaccines based on self-amplifying RNA lipid nanparticles (saRNA LNPs), saRNA transfected Lactobacillus plantarum LNPs, and saRNA transfected Lactobacillus plantarum to neutralize SARS-CoV-2 variants alpha and delta
Source: Sci Rep. 2021 Oct 29;11:21308. doi: 10.1038/s41598-021-00830-5 (PMC8556360; doi:10.1038/s41598-021-00830-5)
Supplement: Supplementary file 2 — Supplementary Information 2. [file 41598_2021_830_MOESM2_ESM.docx]

**Supplementary 2.** The level of INF-γ, TNF-α, IL-4, and IL-10 in the serum of vaccinated mice and recovered COVID-19 patients.

To detect the level of INF-γ, TNF-α, IL-4, and IL-10 in the serum of vaccinated mice, a high binding ELISA plate (Biomat, Italy) was separately coated with anti-mouse and anti-human INF-γ, TNF-α, IL-4, and IL-10 IgG (Southern Biotech) and then 50 μL of serum samples from immunized mice, serum samples from recovered COVID-19 were separately added to wells. After 1 hour incubation at 37 °C, plates were washed with PBS and then 100 μL of secondary antibodies, including anti-human INF-γ-HRP, TNF-α-HRP, IL-4-HRP, and IL-4-HRP (Southern Biotech) were separately added. Then, 50 μL of 3,3′, 5,5′-tetramethylbenzidine was added and after 15 minutes 100 μL of sulfuric acid 1% (Sigma) was added. Finally, the absorbance of each well was read by a Spectrophotometer at 450 nm (BioTek Industries) and then the serum level of INF-γ, TNF-α, IL-4, and IL-10 was quantified by standard curve. From the experiment, we observed the higher secretion of IFN-γ and TNF-α compared with IL-4 and IL-10 in all vaccinated mice, indicating Th1-biased response.

The level of INF-γ, TNF-α, IL-6, and IL-10 in the serum of vaccinated mice and recovered COVID-19 patients.

|  | **INF-γ (ng/ml)** | **TNF-α (ng/ml)** | **IL-4 (pg/ml)** | **IL-10 (pg/ml)** |
| --- | --- | --- | --- | --- |
| **Negative Control** | `0.1±0.005 | 0.1±0.005 | 1±0.05 | 1±0.05 |
| **0.1 μg *Lactobacillus plantarum* LNPs** | 10±0.1* | 8±0.1* | 100±5* | 80±4* |
| **1 μg *Lactobacillus plantarum* LNPs** | 11±0.2* | 9±0.3* | 110±5* | 74±4* |
| **10 μg *Lactobacillus plantarum* LNPs** | 12±0.1* | 10±0.2* | 120±4* | 65±3* |
| **0.1 μg saRNA LNPs** | 11±0.2* | 9±0.1* | 110±4* | 81±5* |
| **1 μg saRNA LNPs** | 12±0.05* | 10±0.1* | 130±5* | 75±4* |
| **10 μg saRNA LNPs** | 13±0.02* | 11±0.2* | 140±5* | 62±3* |
| **10 μg *Lactobacillus plantarum*** | 10±0.1* | 10±0.1* | 120±6* | 72±4* |
| **Recovered COVID-19 patients** | 0.1±0.005 | 0.1±0.005 | 1±0.05 | 1±0.05 |

* indicates significance difference with *P*<0.05 when compared with negative control using one-way ANOVA with n =10 biologically independent mice and n=10 recovered COVID-19 patients.
