# Supplementary material for: The evaluation of novel oral vaccines based on self-amplifying RNA lipid nanparticles (saRNA LNPs), saRNA transfected Lactobacillus plantarum LNPs, and saRNA transfected Lactobacillus plantarum to neutralize SARS-CoV-2 variants alpha and delta
Source: Sci Rep. 2021 Oct 29;11:21308. doi: 10.1038/s41598-021-00830-5 (PMC8556360; doi:10.1038/s41598-021-00830-5)
Supplement: Supplementary file 3 — Supplementary Information 3. [file 41598_2021_830_MOESM3_ESM.docx]

**Supplementary 3.** The full length sequence of saRNA construct used in this study.

5′-GTGAATGATGATGGCGTCAAAAGACGTCGTTCCTACTGCTGCTAGCAGTGAAAATGCTAACAACAATAGTAGTATTAAGTCTCGTCTATTGGCGAGACTCAAGGGTTCAGGTGGGGCTACGTCCCCACCCAACTCGATAAAGATAACCAACCAAGATATGGCTCTGGGGCTGATTGGACAGGTCCCAGCGCCAAAGGCCACATCCGTCGATGTCCCTAAACAACAGAGGGATAGACCACCACGGACTGTTGCCGAAGTTCAACAAAATTTGCGTTGGACTGAGAGACCACAAGACCAGAATGTTAAGACGTGGGATGAGCTTGACCACACAACAAAACAACAGATACTTGATGAACACGCTGAGTGGTTTGATGCCGGTGGCTTAGGTCCAAGTACACTACCCACTAGTCATGAACGGTACACACATGAGAATGATGAAGGCCACCAGGTAAAGTGGTCGGCTAGGGAAGGTGTAGACCTTGGCATATCCGGGCTCACGACGGTGTCTGGGCCTGAGTGGAATATGTGCCCGCTACCACCAGTTGACCAAAGGAGCACGACACCTGCAACTGAGCCCACAATTGGTGACATGATCGAATTCTATGAAGGGCACATCTATCATTATGCTATATACATAGGTCAAGGCAAGACGGTGGGTGTACACTCCCCTCAAGCAGCCTTCTCAATAACGAGGATCACCATACAGCCCATATCAGCTTGGTGGCGAGTCTGTTATGTCCCACAACCAAAACAGAGGCTCACATACGACCAACTCAAAGAATTAGAAAATGAACCATGGCCGTATGCCGCAGTCACGAACAACTGCTTCGAATTTTGTTGCCAGGTCATGTGCTTGGAAGATACTTGGTTGCAAAGGAAGCTCATCTCCTCTGGCCGGTTTTACCACCCGACCCAAGATTGGTCCCGAGACACTCCAGAATTCCAACAAGACAGCAAGTTAGAGATGGTTAGGGATGCAGTGCTAGCCGCTATAAATGGGTTGGTGTCGCGGCCATTTAAAGATCTTCTGGGTAAGCTCAAACCCTTGAACGTGCTTAACTTACTTTCAAACTGTGATTGGACGTTCATGGGGGTCGTGGAGATGGTGGTCCTCCTTTTAGAACTCTTTGGAATCTTTTGGAACCCACCTGATGTTTCCAACTTTATAGCTTCACTCCTGCCAGATTTCCATCTACAGGGCCCCGAGGACCTTGCCAGGGATCTCGTGCCAATAGTATTGGGGGGGATCGGCTTAGCCATAGGATTCACCAGAGACAAGGTAAGTAAGATGATGAAGAATGCTGTTGATGGACTTCGTGCGGCAACCCAGCTCGGTCAATATGGCCTAGAAATATTCTCATTACTAAAGAAGTACTTCTTCGGTGGTGATCAAACAGAGAAAACCCTAAAAGATATTGAGTCAGCAGTTATAGATATGGAAGTACTATCATCTACATCAGTGACTCAGCTCGTGAGGGACAAACAGTCTGCACGGGCTTATATGGCCATCTTAGATAATGAAGAAGAAAAGGCAAGGAAATTATCTGTCAGGAATGCCGACCCACACGTAGTATCCTCTACCAATGCTCTCATATCCCGGATCTCAATGGCTAGGGCTGCATTGGCCAAGGCTCAAGCTGAAATGACCAGCAGGATGCGTCCTGTGGTCATTATGATGTGTGGGCCCCCTGGTATAGGTAAAACCAAGGCAGCAGAACATCTGGCTAAACGCCTAGCCAATGAGATACGGCCTGGTGGTAAGGTTGGGCTGGTCCCACGGGAGGCAGTGGATCATTGGGATGGATATCACGGAGAGGAAGTGATGCTGTGGGACGACTATGGAATGACAAAGATACAGGAAGACTGTAATAAACTGCAAGCCATAGCCGACTCAGCCCCCCTAACACTCAATTGTGACCGAATAGAAAACAAGGGAATGCAATTTGTGTCTGATGCTATAGTCATCACCACCAATGCTCCTGGCCCAGCCCCAGTGGACTTTGTCAACCTCGGGCCTGTTTGCCGAAGGGTGGACTTCCTTGTGTATTGCACGGCACCTGAAGTTGAACACACGAGGAAAGTCAGTCCTGGGGACACAACTGCACTGAAAGACTGCTTCAAGCCCGATTTCTCACATCTAAAAATGGAGTTGGCTCCCCAAGGGGGCTTTGATAACCAAGGGAATACCCCGTTTGGTAAGGGTGTGATGAAGCCCACCACCATAAACAGGCTGTTAATCCAGGCTGTAGCCTTGACGATGGAGAGACAGGATGAGTTCCAACTCCAGGGGCCTACGTATGACTTTGATACTGACAGAGTAGCTGCGTTCACGAGGATGGCCCGAGCCAACGGGTTGGGTCTCATATCCATGGCCTCCCTAGGCAAAAAGCTACGCAGTGTCACCACTATTGAAGGATTAAAGAATGCTCTATCAGGCTATAAAATATCAAAATGCAGTATACAATGGCAGTCAAGGGTGTACATTATAGAATCAGATGGTGCCAGTGTACAAATCAAAGAAGACAAGCAAGCTTTGACCCCTCTGCAGCAGACAATTAACACGGCCTCACTTGCCATCACTCGACTCAAAGCAGCTAGGGCTGTGGCATACGCTTCATGTTTCCAGTCCGCCATAACTACCATACTACAAATGGCGGGATCTGCGCTCGTTATTAATCGAGCGGTCAAGCGTATGTTTGGTACCCGTACAGCAGCCATGGCATTAGAAGGACCTGGGAAAGAACATAATTGCAGGGTCCATAAGGCTAAGGAAGCTGGAAAGGGGCCCATAGGTCATGATGACATGGTAGAAAGGTTTGGCCTATGTGAAACTGAAGAGGAGGAGAGTGAGGACCAAATTCAAATGGTACCAAGTGATGCCGTCCCAGAAGGAAAGAACAAAGGCAAGACCAAAAAGGGACGTGGTCGCAAAAATAACTATAATGCATTCTCTCGCCGTGGTCTGAGTGATGAAGAATATGAAGAGTACAAAAAGATCAGAGAAGAAAAGAATGGCAATTATAGTATACAAGAATACTTGGAGGACCGCCAACGATATGAGGAAGAATTAGCAGAGGTACAGGCAGGTGGTGATGGTGGCATAGGAGAAACTGAAATGGAAATCCGTCACAGGGTCTTCTATAAATCCAAGAGTAAGAAACACCAACAAGAGCAACGGCGACAACTTGGTCTAGTGACTGGATCAGACATCAGAAAACGTAAGCCCATTGACTGGACCCCGCCAAAGAATGAATGGGCAGATGATGACAGAGAGGTGGATTATAATGAAAAGATCAATTTTGAAGCTCCCCCGACACTATGGAGCCGAGTCACAAAGTTTGGATCAGGATGGGGCTTTTGGGTCAGCCCGACAGTGTTCATCACAACCACACATGTAGTGCCAACTGGTGTGAAAGAATTCTTTGGTGAGCCCCTATCTAGTATAGCAATCCACCAAGCAGGTGAGTTCACACAATTCAGGTTCTCAAAGAAAATGCGCCCTGACTTGACAGGTATGGTCCTTGAAGAAGGTTGCCCTGAAGGGACAGTCTGCTCAGTCCTAATTAAACGGGATTCGGGTGAACTACTTCCGCTAGCCGTCCGTATGGGGGCTATTGCCTCCATGAGGATACAGGGTCGGCTTGTCCATGGCCAATCAGGGATGTTACTGACAGGGGCCAATGCAAAGGGGATGGATCTTGGCACTATACCAGGAGACTGCGGGGCACCATACGTCCACAAGCGCGGGAATGACTGGGTTGTGTGTGGAGTCCACGCTGCAGCCACAAAGTCAGGCAACACCGTGGTCTGCGCTGTACAGGCTGGAGAGGGCGAAACCGCACTAGAAGGTGGAGACAAGGGGCATTATGCCGGCCACGAGATTGTGAGGTATGGAAGTGGCCCAGCACTGTCAACTAAAACAAAATTCTGGAGGTCCTCCCCAGAACCACTGCCCCCCGGAGTATATGAGCCAGCATACCTGGGGGGCAAGGACCCCCGTGTACAGAATGGCCCATCCCTACAACAGGTACTACGTGACCAACTGAAACCCTTTGCGGACCCCCGCGGCCGCATGCCTGAGCCTGGCCTACTGGAGGCTGCGGTTGAGACTGTAACATCCATGTTAGAACAGACAATGGATACCCCAAGCCCGTGGTCTTACGCTGATGCCTGCCAATCTCTTGACAAAACTACTAGTTCGGGGTACCCTCACCATAAAAGGAAGAATGATGATTGGAATGGCACCACCTTCGTTGGAGAGCTCGGTGAGCAAGCTGCACACGCCAACAATATGTATGAGAATGCTAAACATATGAAACCCATTTACACTGCAGCCTTAAAAGATGAACTAGTCAAGCCAGAAAAGATTTATCAAAAAGTCAAGAAGCGTCTACTATGGGGCGCCGATCTCGGAACAGTGGTCAGGGCCGCCCGGGCTTTTGGCCCATTTTGTGACGCTATAAAATCACATGTCATCAAATTGCCAATAAAAGTTGGCATGAACACAATAGAAGATGGCCCCCTCATCTATGCTGAGCATGCTAAATATAAGAATCATTTTGATGCAGATTATACAGCATGGGACTCAACACAAAATAGACAAATTATGACAGAATCCTTCTCCATTATGTCGCGCCTTACGGCCTCACCAGAATTGGCCGAGGTTGTGGCCCAAGATTTGCTAGCACCATCTGAGATGGATGTAGGTGATTATGTCATCAGGGTCAAAGAGGGGCTGCCATCTGGATTCCCATGTACTTCCCAGGTGAACAGCATAAATCACTGGATAATTACTCTCTGTGCACTGTCTGAGGCCACTGGTTTATCACCTGATGTGGTGCAATCCATGTCATATTTCTCATTTTATGGTGATGATGAGATTGTGTCAACTGACATAGATTTTGACCCAGCCCGCCTCACTCAAATTCTCAAGGAATATGGCCTCAAACCAACAAGGCCTGACAAAACAGAAGGACCAATACAAGTGAGGAAAAATGTGGATGGACTGGTCTTCTTGCGGCGCACCATTTCCCGTGATGCGGCAGGGTTCCAAGGCAGGTTAGATAGGGCTTCGATTGAACGCCAAATCTTCTGGACCCGCGGGCCCAATCATTCAGATCCATCAGAGACTCTAGTGCCACACACTCAAAGAAAAATACAGTTGATTTCACTTCTAGGGGAAGCTTCACTCCATGGTGAGAAATTTTACAGAAAGATTTCCAGCAAGGTCATACATGAAATCAAGACTGGTGGATTGGAAATGTATGTCCCAGGATGGCAGGCCATGTTCCGCTGGATGCGCTTCCATGACCTCGGATTGTGGACAGGAGATCGCGATCTTCTGCCCGAATTCGTAAATGATGATGGCGTCTAAtaacctgaatggactacgacatagtctagtccgccaagatgtttgtttttcttgttttattgccactagtctctagtcagtgtgttaatcttacaaccagaactcaattaccccctgcatacactaattctttcacacgtggtgtttattaccctgacaaagttttcagatcctcagttttacattcaactcaggacttgttcttacctttcttttccaatgttacttggttccatgctatacatgtctctgggaccaatggtactaagaggtttgataaccctgtcctaccatttaatgatggtgtttattttgcttccattgagaagtctaacataataagaggctggatttttggtactactttagactcgaagacccagtccctacttattgttaataacgctactaatgttgttattaaagtctgtgaatttcaattttgtaatgatccatttttggatgtttattaccacaaaaacaacaaaagttggatgaaaagtgagttcagagtttattctagtgcgaataattgcacttttgaatatgtctctcagccttttcttatggaccttgaaggaaaacagggtaatttcaaaaatcttagggaatttgtgtttaagaatattgatggttattttaaaatatattctaagcacacgcctattaatttagtgcgtgatctccctcagggtttttcggctttagaaccattggtagatttgccaataggtattaacatcactaggtttcaaactttacttgctttacatagaagttatttgactcctggtgattcttcttcaggttggacagctggtgctgcagcttattatgtgggttatcttcaacctaggacttttctattaaaatataatgaaaatggaaccattacagatgctgtagactgtgcacttgaccctctctcagaaacaaagtgtacgttgaaatccttcactgtagaaaaaggaatctatcaaacttctaactttagagtccaaccaacagaatctattgttagatttcctaatattacaaacttgtgcccttttggtgaagtttttaacgccaccagatttgcatctgtttatgcttggaacaggaagagaatcagcaactgtgttgctgattattctgtcctatataattccgcatcattttccacttttaagtgttatggagtgtctcctactaaattaaatgatctctgctttactaatgtctatgcagattcatttgtaattagaggtgatgaagtcagacaaatcgctccagggcaaactggaaagattgctgattataattataaattaccagatgattttacaggctgcgttatagcttggaattctaacaatcttgattctaaggttggtggtaattataattaccggtatagattgtttaggaagtctaatctcaaaccttttgagagagatatttcaactgaaatctatcaggccggtagcacaccttgtaatggtgttcaaggttttaattgttactttcctttacaatcatatggtttccaacccactaatggtgttggttaccaaccatacagagtagtagtactttcttttgaacttctacatgcaccagcaactgtttgtggacctaaaaagtctactaatttggttaaaaacaaatgtgtcaatttcaacttcaatggtttaacaggcacaggtgttcttactgagtctaacaaaaagtttctgcctttccaacaatttggcagagacattgctgacactactgatgctgtccgtgatccacagacacttgagattcttgacattacaccatgttcttttggtggtgtcagtgttataacaccaggaacaaatacttctaaccaggttgctgttctttatcagggtgttaactgcacagaagtccctgttgctattcatgcagatcaacttactcctacttggcgtgtttattctacaggttctaatgtttttcaaacacgtgcaggctgtttaataggggctgaacatgtcaacaactcatatgagtgtgacatacccattggtgcaggtatatgcgctagttatcagactcagactaattctcgtcggcgggcacgtagtgtagctagtcaatccatcattgcctacactatgtcacttggtgcagaaaattcagttgcttactctaataactctattgccatacccacaaattttactattagtgttaccacagaaattctaccagtgtctatgaccaagacatcagtagattgtacaatgtacatttgtggtgattcaactgaatgcagcaatcttttgttgcaatatggcagtttttgtacacaattaaaccgtgctttaactggaatagctgttgaacaagacaaaaacacccaagaagtttttgcacaagtcaaacaaatttacaaaacaccaccaattaaagattttggtggttttaatttttcacaaatattaccagatccatcaaaaccaagcaagaggtcatttattgaagatctacttttcaacaaagtgacacttgcagatgctggcttcatcaaacaatatggtgattgccttggtgatattgctgctagagacctcatttgtgcacaaaagtttaacggccttactgttttgccacctttgctcacagatgaaatgattgctcaatacacttctgcactgttagcgggtacaatcacttctggttggacctttggtgcaggtgctgcattacaaataccatttgctatgcaaatggcttataggtttaatggtattggagttacacagaatgttctctatgagaaccaaaaattgattgccaaccaatttaatagtgctattggcaaaattcaagactcactttcttccacagcaagtgcacttggaaaacttcaagatgtggtcaaccaaaatgcacaagctttaaacacgcttgttaaacaacttagctccaattttggtgcaatttcaagtgttttaaatgatatcctttcacgtcttgacaaagttgaggctgaagtgcaaattgataggttgatcacaggcagacttcaaagtttgcagacatatgtgactcaacaattaattagagctgcagaaatcagagcttctgctaatcttgctgctactaaaatgtcagagtgtgtacttggacaatcaaaaagagttgatttttgtggaaagggctatcatcttatgtccttccctcagtcagcacctcatggtgtagtcttcttgcatgtgacttatgtccctgcacatgaaaagaacttcacaactgctcctgccatttgtcatgatggaaaagcacactttcctcgtgaaggtgtctttgtttcaaatggcacacactggtttgtaacacaaaggaatttttatgaaccacaaatcattactacagacaacacatttgtgtctggtaactgtgatgttgtaataggaattgtcaacaacacagtttatgatcctttgcaacctgaattagactcattcaaggaggagttagataaatattttaagaatcatacatcaccagatgttgatttaggtgacatctctggcattaatgcttcagttgtaaacattcaaaaagaaattgaccgcctcaatgaggttgccaagaatttaaatgaatctctcatcgatctccaagaacttggaaagtatgagcagtatataaaatggccatggtacatttggctaggttttatagctggcttgattgccatagtaatggtgacaattatgctttgctgtatgaccagttgctgtagttgtctcaagggctgttgttcttgtggatcctgctgcaaatttgatgaagacgactctgagccagtgctcaaaggagtcaaattacattacacataatgttgtaatatgaaatgtgggcatcatattcatttaattaggtttaattaggtttaatttgatgttAAAAA

-3′

The saRNA construct has different parts, including: 1) 5′ UTR (GenBank accession number: NC_001959.2), 2) Norovirus GI (GenBank accession number: NC_001959.2), 3) noncoding segment (GenBank accession number: NC_001959.2), 4) S-protein (GenBank accession number: MZ571142.1), 5) 3′ UTR (GenBank accession number: NC_001959.2), and 6) polyA tail.
