# Supplementary material for: The evaluation of novel oral vaccines based on self-amplifying RNA lipid nanparticles (saRNA LNPs), saRNA transfected Lactobacillus plantarum LNPs, and saRNA transfected Lactobacillus plantarum to neutralize SARS-CoV-2 variants alpha and delta
Source: Sci Rep. 2021 Oct 29;11:21308. doi: 10.1038/s41598-021-00830-5 (PMC8556360; doi:10.1038/s41598-021-00830-5)
Supplement: Supplementary file 4 — Supplementary Information 4. [file 41598_2021_830_MOESM4_ESM.docx]

**Supplementary 4.** The confirmation of *Lactobacillus plantarum* transfection by real-time PCR.

*Lactobacillus plantarum* was transfected with plasmids by electroporation. After bacterial selection, they were cultured and the plasmids were purified and then 1 ng of purified plasmid, 0.5 μM primers (forward: 5′-CTATCAGGCCGGTAGCACAC-3′ and reverse: 5′-ACACCTGTGCCTGTTAAACCA-3′) and 2 U of Mastermix qPCR SYBER (Invitrogen, UK) were added and amplified under the following conditions: 95 °C for 1 min; 35 cycles of 95 °C for 10 s, 55 °C for 30 s, and 72 °C for 10 s; 72 °C for 5 min by an ABI real-time PCR system (Applied Biosystem, StepOne plus). The 16S rRNA gene was used as an internal control and delta-delta CT formula was used to evaluate the relative gene expression. As seen, high number of desired plasmid were detected in saRNA transfected *Lactobacillus plantarum.*
