# Supplementary material for: The evaluation of novel oral vaccines based on self-amplifying RNA lipid nanparticles (saRNA LNPs), saRNA transfected Lactobacillus plantarum LNPs, and saRNA transfected Lactobacillus plantarum to neutralize SARS-CoV-2 variants alpha and delta
Source: Sci Rep. 2021 Oct 29;11:21308. doi: 10.1038/s41598-021-00830-5 (PMC8556360; doi:10.1038/s41598-021-00830-5)
Supplement: Supplementary file 5 — Supplementary Information 5. [file 41598_2021_830_MOESM5_ESM.docx]

**Supplementary 5.** The invitro expression of S-protein**.**

HEK293T/17 cells were cultured at 37 °C for 48 hours and separately incubated with 10 µg saRNA LNPs, 10 µg transfected *Lactobacillus plantarum* LNPs, and 10 µg transfected *Lactobacillus plantarum*. After 24 hours, the expression of S-protein was confirmed by real-time PCR and ELISA. For real-time PCR, RiboEx total RNA extraction buffer (GeneAll, South Korea) was used to extract total RNA. Then, 1 ng of total RNA was mixed with 5µL of Mastermix First-Strand Synthesis kit (Takara Bio Inc, USA) and incubated for 30 minutes at 50 °C and then for 30 minutes at 95 °C to inactivate RT enzyme. Then, 5µL of cDNA, 5 µL of primers (forward: 5′- CTATCAGGCCGGTAGCACAC -3′ and reverse: 5′- ACACCTGTGCCTGTTAAACCA -3′) and 10µL of Mastermix qPCR SYBER (Invitrogen, UK) were mixed and amplified under the following conditions: 95 °C for 1 min; 40 cycles of 95 °C for 10 s, 55 °C for 30 s, and 72 °C for 10 s; 72 °C for 5 min by an ABI real-time PCR system (Applied Biosystem, StepOne plus). The GAPDH gene was used as an internal control and delta-delta CT formula was used to evaluate the relative gene expression. For ELISA, a high binding ELISA plates (Biomat, Italy) were coated with anti-S-protein SARS-CoV-2 IgG (Sigma-Aldrich) at 1 mg/mL overnight at 4°C. The plates were washed 3 times with PBS and blocked with 2% BSA (Sigma-Aldrich) and 3% sucrose (Sigma-Aldrich) at 4 °C overnight. Then, treated HEK293T/17 cells were lyzed by lysing buffer (Thermo Fisher Scientific) and centrifuged at 5000 RPM for 5 minutes. Then, 100 µL of supernatant was added to the ELISA plate. The plates were incubated at 37 °C for 2 hours and then were washed 3 times with PBS. After incubation and washing, they were separately incubated with HRP-conjugated antibodies (anti-S-protein SARS-CoV-2-HRP IgG(Sigma-Aldrich) with 1:5000 at 37 °C for one hour. After washing, 100 µL TMB substrate (Sigma-Aldrich) was added and incubated at 37°C for 15 minutes. Then, 100 µL of sulfuric acid (Sigma) was added and the optical density of each well was measured at 450 nm by a spectrophotometer (BioTek Industries). To quantify the level of S-protein, a standard curve was used.
