# Supplementary material for: The evaluation of novel oral vaccines based on self-amplifying RNA lipid nanparticles (saRNA LNPs), saRNA transfected Lactobacillus plantarum LNPs, and saRNA transfected Lactobacillus plantarum to neutralize SARS-CoV-2 variants alpha and delta
Source: Sci Rep. 2021 Oct 29;11:21308. doi: 10.1038/s41598-021-00830-5 (PMC8556360; doi:10.1038/s41598-021-00830-5)
Supplement: Supplementary file 6 — Supplementary Information 6. [file 41598_2021_830_MOESM6_ESM.docx]

**Supplementary 6.** Biodistribution assay.

BALB/c mice aged 6–8 weeks were orally administered with single dose of 10µg saRNA LNPs, 10 µg transfected *Lactobacillus plantarum* LNPs, and 10 µg transfected *Lactobacillus plantarum*. At two time intervals (1 and 24 hours), mice were sacrificed and sampled from major organs or tissues, such as small intestine, large intestine, liver, blood, spleen, and muscle. Then, the concentration of S-protein was quantified by ELISA. A high binding ELISA plates (Biomat, Italy) were coated with anti-S-protein SARS-CoV-2 IgG (Sigma-Aldrich) at 1 mg/mL overnight at 4°C. The plates were washed 3 times with PBS and blocked with 2% BSA (Sigma-Aldrich) and 3% sucrose (Sigma-Aldrich) at 4 °C overnight. Then, treated HEK293T/17 cells were lyzed by lysing buffer (Thermo Fisher Scientific) and centrifuged at 5000 RPM for 5 minutes. Then, 100 µL of supernatant was added to the ELISA plate. The plates were incubated at 37 °C for 2 hours and then were washed 3 times with PBS. After incubation and washing, they were separately incubated with HRP-conjugated antibodies (anti-S-protein SARS-CoV-2-HRP IgG(Sigma-Aldrich) with 1:5000 at 37 °C for one hour. After washing, 100 µL TMB substrate (Sigma-Aldrich) was added and incubated at 37°C for 15 minutes. Then, 100 µL of sulfuric acid (Sigma) was added and the optical density of each well was measured at 450 nm by a spectrophotometer (BioTek Industries). To quantify the level of S-protein, a standard curve was used.
